# Natural Non-Trasgenic Animal Models for Research in Alzheimer’s Disease

**DOI:** 10.2174/156720509787602834

**Published:** 2009-04

**Authors:** Manuel Sarasa, Pedro Pesini

**Affiliations:** Araclon Biotech S.L., 50004 Zaragoza, Spain

**Keywords:** Chick embryo, dog, dolphin, rabbit, rat, APP, Aβ.

## Abstract

The most common animal models currently used for Alzheimer disease (AD) research are transgenic mice that express a mutant form of human Aβ precursor protein (APP) and/or some of the enzymes implicated in their metabolic processing. However, these transgenic mice carry their own APP and APP-processing enzymes, which may interfere in the production of different amyloid-beta (Aβ) peptides encoded by the human transgenes. Additionally, the genetic backgrounds of the different transgenic mice are a possible confounding factor with regard to crucial aspects of AD that they may (or may not) reproduce. Thus, although the usefulness of transgenic mice is undisputed, we hypothesized that additional relevant information on the physiopathology of AD could be obtained from other natural non-transgenic models. We have analyzed the chick embryo and the dog, which may be better experimental models because their enzymatic machinery for processing APP is almost identical to that of humans. The chick embryo is extremely easy to access and manipulate. It could be an advantageous natural model in which to study the cell biology and developmental function of APP and a potential assay system for drugs that regulate APP processing. The dog suffers from an age-related syndrome of cognitive dysfunction that naturally reproduces key aspects of AD including Aβ cortical pathology, neuronal degeneration and learning and memory disabilities. However, dense core neuritic plaques and neurofibrillary tangles have not been consistently demonstrated in the dog. Thus, these species may be natural models with which to study the biology of AD, and could also serve as assay systems for Aβ-targeted drugs or new therapeutic strategies against this devastating disease.

## INTRODUCTION

The ethics of scientific research obliges to reduce the use of laboratory animals to the minimum possible. Nevertheless, studies in animal models are still necessary to understand the mechanisms of many human diseases and to try new therapeutic strategies. In any case, experiments with animals must always be approved by ethical committees and to enforce general and local normatives (see for example European and Spanish legislations on animal handling 86/609/EU and Real Decreto 1201/2005, respectively).

Rodents in general, and the rat and the mouse in particular, are the animals most frequently used as experimental model organisms for a great variety of human diseases. The short reproductive cycle of these small mammals (21 days of gestation), their high birth rate, and their small size make them relatively easy to maintain and manage. Recent developments in genetic engineering and biotechnology have led to the production of a diverse selection of transgenic animals, mainly mice. These transgenic animals (bearing an exogenous gene, usually from human origin), together with other agenics or knockouts (in which a particular gene has been blocked or eliminated) have been very useful in studying the presumptive roles and mechanisms involving the proteins coded by the transgene or the knockout gene of interest. Translational research has brought particular these technologies to the field of clinical investigation, with the production of inbred animal lines that serve as models for human diseases. Numerous knockout and transgenic animal models have been developed to explore particular aspects of Alzheimer’s disease. 

Suppression of genes that encode proteins involved in Alzheimer’s, such as APP, apolipoprotein E (ApoE), β-secretase, or presenilins, has helped us to anticipate the effects of presumptive new molecules designed to regulate these proteins. For example, presenilin knockout mice develop striking neurodegeneration of the cerebral cortex and worsening impairments of memory and synaptic function with increasing age, which call for caution in the therapeutic use of γ-secretase inhibitors [1]. In contrast, β-secretase knockout mice are perfectly viable [2]. The absence of lethal phenotypes for β-secretase knockouts, which cannot generate Aβ peptide, feeds the hope of success for therapeutic strategies based on the use of β-secretase inhibitors or aimed directly at the blockade or elimination of Aβ peptide. Nevertheless, APP knockout mice present overt neurological deficiencies which can be reverted if the endogenous APP locus is replaced by a gene-targeted allele (knock-in mice) that exclusively express the secreted APP ectodomain [3, 4]. In addition, functional redundancy between APP and other APP-like proteins has been reported [5]. The development of transgenic mice lines with mutated human APP genes has been particularly enlightening, since these animals develop Aβ deposits in their brains and reproduce some of the neurological symptoms of AD. However, most of these species lack essential features of AD despite the increasingly complex genotypes that are being engineered to approach a more complete disease phenotype [6-14]. The heterogeneity of different transgenics should be considered a possible confounding factor that seeds doubts when observations made in humans must be interpreted in the light of results obtained in these models. Uncertainty is also unavoidable when the efficacy of new AD therapies must be forecasted from trials in transgenic mice.

Thus, although the usefulness of transgenic mice is undisputed, we hypothesized that additional relevant information regarding the pathological mechanisms of AD could be obtained from natural non-transgenic models. In the following pages we will refer to some of the results obtained in our laboratory that led us to propose new wild type animal models for the analysis of relevant aspects of AD. We have designed and produced highly specific and sensitive antibodies against the two major isoforms of the Aβ amyloid to aid in this research, and are currently exploring the use of these Aβ immunogens in the search for premorbid biomarkers and immunotherapeutic strategies. 

## THE RAT

The rat is the most frequently utilized experimental animal in neuroscience, although with regard to AD the use of transgenic mice has been increasing. Nevertheless, an enormous amount of work has been produced in the rat, and important advances in the understanding of APP processing— including the studies central to the discovery of neprilysin [15] and insulin degrading enzyme (IDE) [16] as the main proteases implicated in Aβ degradation— have been obtained from experiments conducted in rats. The rat has also been used to study the effects of induced cortical Aβ accumulation on the cholinergic, noradrenergic, and serotonergic systems [17], as well as the influence of diet and age on the effects of intracerebral beta-amyloid injection [18]. However, it should be emphasized that the primary structures of the Aβ peptide in rats (and mice) differ from their human counterpart at three amino acid residues, and that the complete Aβ-peptide profile produced by the processing of APP in these rodents’ also reveals major differences compared to that of humans [19].

In our laboratory, the rat was chosen to study the kinetics of the expression of the different APP isoforms in response to axotomy [20]. We found increased levels of APP 695, 714, 751, and 770 mRNAs after facial or hypoglossal nerve axotomy of the parent ipsilateral motor neurons. The increase was gradual, reaching maximal values 7 days after axotomy, and returning to normal values by 60 days after the lesion. Increased APP immunostaining was also detected in the chromatolytic motor neurons, but not in the reactive astrocytes surrounding axotomized neurons. These results suggested a possible neuroprotective role for APP that could also be involved in a variety of cell processes that remain poorly understood [21-23]. In particular, APP has been implicated in neurotrophic, synaptogenic, and growth-promoting mechanisms which led us to investigate the expression of this protein during the rat embryogenesis [24]. Using oligonucleotides recognizing each of the four major APP (APP-695, APP-714, APP-751, and APP-770) mRNAs, we found that each APP displays a specific temporal and spatial expression pattern. The prototype isoform APP-695 appears early in embryogenesis within cells actively implicated in morphogenetic events, such as the mesodermal cells that invaginate at the level of the primitive streak, and is later restricted to the neurectodermal derivatives (neural tube, neural crest, and neurogenic placode). By contrast, expression of the longest isoform, APP-770, occurs later and is restricted to mesodermal and endodermal derivatives. The isoforms of 714 and 751 amino acids are expressed still later in the course of prenatal development, and appear to be distributed ubiquitously.

Another wild type rodent used for research in AD is the guinea pig [25]. Beta-amyloid peptide sequence expressed by guinea pigs is identical to the human sequence, and appears to represent a more physiological model with which to examine *in vivo* the long-term effects of experimental manipulations on APP processing. Additionally, APP processing in guinea pig primary neuronal cultures is demonstrably similar to analogous cultures of human origin [25]. However, the use of these animals is limited because they do not develop the pathological features of the disease (senile plaques and neurofibrillary tangles) and there are not well-established behavioral tests for this species.

## THE RABBIT

As in guinea pigs, the rabbit Aβ peptide sequence is identical to human [26] and does not present AD pathology spontaneously. However, when copper is added to the diet of cholesterol-fed rabbits, they develop cortical amyloid deposits and up to twelve other pathological markers seen in AD, including retardation of the ability to learn a difficult task [27]. This finding suggests that copper (but apparently not other heavy metals like aluminum or zinc) may influence the progression of Alzheimer's disease by reducing clearance of Aβ from the brain [28, 29]. This finding also emphasized the usefulness of the rabbit model, whose validity is being extended even to treatment modalities [30-32].

## THE CHICK EMBRYO

Our studies regarding the prenatal expression of different APPs in the rat embryo [24] were coincident in time with the publication of reports on the lack of substantial phenotypic differences in APP knockout mice [5]. This led us to explore the chick embryo as a possible model for the investigation of presumptive APP functions during prenatal development. The chick embryo is readily available and easy to work with, and our laboratory has extensive experience with this model. Before engaging in experiments on the functional blocking of APP, we first needed to know the sequence of the APP gene in the chicken. We isolated the total RNA from the chick embryo and cloned the cDNA [33]. We found two of the major APP isoforms, APP-751, which contains the KPI domain, and APP-695. The APP-770 isoform, which is the longest isoform and contains the OX-2 domain, was absent or minimally expressed. Interestingly, the chick and human gene sequences are almost identical, with 93% amino acid identity and 96% similarity. The KPI domain of APP-751 has 52 identical residues out of 56 (93% identity), and three of the four different residues belong to the same group of amino acids (98% similarity). The 40–42 amino acid residues of the Aβ domain are identical, as are the last 101 C-terminal amino acids of the APP sequence (corresponding to the Aβ, transmembrane and intracytoplasmic domains). We also established through whole-mount *in situ* hybridization histochemistry and immunohistochemistry^1^ that chicken APP expression parallels mammalian APP expression both temporally and topographically [32]. After the 3^rd^ day of embryonic development, APP-695 mRNA expression levels were much higher than APP-751 expression levels in the neuroectodermal derivatives, including the neural tube; the sensory, autonomic and cranial ganglia; the retina; and the otic vesicle. Furthermore, the chick embryo expresses the genes that encode the main proteases implicated in the production of beta amyloid protein, including BACE-1, BACE-2, presenilin-1, presenilin-2, and nicastrin; Aβ1-42 is the major Aβ peptide produced during chick embryogenesis [33]. Neprilysin, the main Aβ-degrading enzyme, and ADAM-17, a protease implicated in the non-amyloidogenic processing of APP, are also expressed in chick embryos. Thus, we propose the chick embryo as a suitable natural model in which to study the cell biology and developmental function of beta-amyloid precursor protein, and a potential assay system for drugs that regulate beta-amyloid precursor protein processing. But do chicks suffer any Alzheimer-like pathology? We have explored whether relatively aged chickens might develop brain amyloid pathology, and have obtained negative results. However, our access has been limited to the brains of chicken up to 10 years old; these individuals appear to be too young to develop amyloid deposits, since the chicken lifespan reaches approximately 30 years [34]. In fact, the brains of 10-year-old chickens do not appear to differ from the brains of chickens between 2 and 14 months old.

## THE DOG

The dog has been pointed out as an especially appropriate model for the study of human brain aging and neurodegenerative diseases. The numerous domestic dog breeds exhibit greater diversity in body size than any other terrestrial vertebrate, and geneticists are taking advantage of this diversity to investigate the genetic basis of size and its relationship to lifespan [35-37]. Canines have evolved phylogenetically in close proximity to humans, making them unusually skilled at reading human social and communicative behavior— even more so than our nearest primate relatives [38, 39]. Provided that ethical normative is enforced, individuals of every age are available for study among the immense population of pet dogs. Canine neurology, including behavioral medicine, is state of the art among veterinary clinical sciences, and access to longitudinal records is already possible in many cases [40, 41]. Dogs are frequently used for the preclinical development of a wide variety of drugs (see for example [42, 43]). Regarding AD, almost every current treatment strategy has been tried out in the dog, from cholinergic agonists to antioxidant and mitochondrial enzymatic cofactors [44-49]. Increasing interest in this model is based on the fact that the dog may naturally develop an age-related cognitive dysfunction that reproduces several aspects of AD [47, 50-53]. Thus, numerous studies with dog cohorts that were submitted to several behavioral paradigms have revealed subsets of aged dogs that had learning and memory impairments [46, 54-57]. This cognitive damage correlated with the extent of Aβ deposits in the cerebral cortex [58-60]. Interestingly, a similar correlation has been reported in domestic pet dogs, based on cognitive status evaluated from questionnaires that were answered by their caregivers [61-63]. Most of these tests for evaluation of the cognitive status assess four main behavioural categories: disorientation (on daily walks or at home), interaction with owners (lost of interest in greeting or playing; lost of response to incitation), sleep/awake cycle (wander and/or bark during night) and lost of the house training habits. It has been reported that 28% of 11-12 year old dogs present impairments in one or more of these categories, of which 10% have impairments in two or more categories and are considered severely impaired. Among 15-16 year old dogs, 68% have impairment in one or more category, of which 35% have impairments in two or more categories [64, 65].

**Fig (1):**
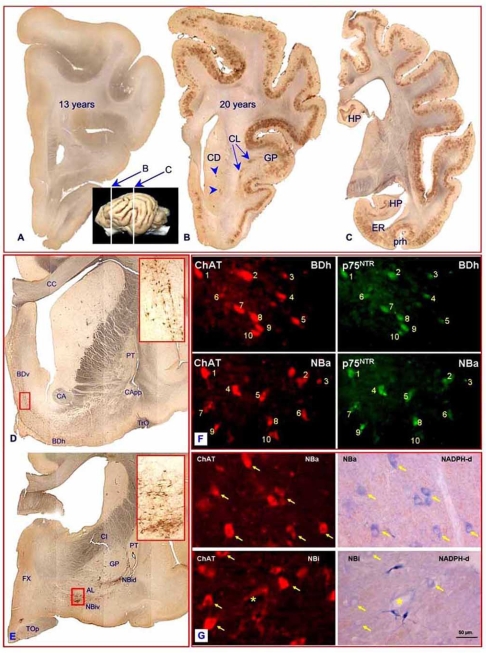
(1A-G). The canine model of Alzheimer’s disease. (**A-C**) Cortical Aβ pathology in the dog. (**A**) Brain section from a 13 years old Pekinese dog without Aβ deposits and (**B-C**) from a 20 years old individual of the same breed with extensive Aβ deposits. The three sections were stained in the same batch with 6E10 monoclonal antibody (Signet Lab. Dedham, USA). The level of **B** and **C** section is marked over the lateral view of the brain (inset). The earliest and more consistently affected areas in the dog were the prefrontal cortex, including the gyrus proreus (GP), the entorhinal cortex (ER) and the hippocampus (HP). However in severe cases as the one shown here (**B** and **C**), amyloid deposits spread throughout the cortex and can be found also in the caudate (arrowheads) and the claustrum (CL). (**D-E**) the anatomical distribution of the neurons expressing the low affinity nerve growth factor receptor (p75_NTR_) in the basal forebrain of the dog. Section in **D** is at the level of the vertical and horizontal diagonal band (BDv and BDh, respectively); section in **E** is at the level of the dorsal and ventral subdivision of the intermediate part of nucleus basalis of Meynert (NBid and NBiv, respectively). Framed area in **D** and **E** are enlarged in the corresponding inset. (**F**) Double immunofluorescence staining showing that practically all magnocellular cholinergic neurons (red staining) in the basal forebrain co-express p75_NTR_ (green staining). (**G**) Co-expression of choline acetyl transferase (ChAT, red immunofluorescence) and NADPH-D (blue staining, bright field illumination) in most (~80%) of the basal magnocellular neurons in the anterior and intermediate parts of the dog (NBa and NBi respectively; yellow arrows). These images were presented at the 17th ECVIM-CA congress and 9th ESVCP congress. Budapest, September 2007. (Suárez M-L, Insua D, Corredoira A, Bernedo V, Santamarina G, and Pesini P. The basal forebrain cholinergic neurons in aged dogs with and without cortical amyloid pathology).

Histopathological studies on this putative canine model of AD showed that the earliest and more consistently affected areas were in the prefrontal cortex, including the gyrus proreus and the hippocampus [59, 66, 67]. Deposition of Aβ in the dog appears to progress from deep to superficial cortical layers, and from diffuse patches to denser and better-delimited plaques [68]. However, dense core neuritic plaques and neurofibrillary tangles have not been consistently demonstrated in the dog [69-72]. Aβ deposits do not appear in every aged dog, as might be expected if they were merely a consequence of normal aging (Fig. **1A-C**). Instead, as occurs in humans, exogenous and/or genetic factors may determine the susceptibility of individual dogs toward developing amyloid pathology at a given age [73, 74]. Nevertheless, in a recent study we have found Aβ angiopathy and a moderate to heavy Aβ burden in two aged dogs, although they have no apparent cognitive symptoms. This finding is reminiscent of similar reports of extensive Aβ deposits in human brains that had not been diagnosed with dementia (reviewed by [75]). It poses the possibility that, as occurs in AD, the development of neuropathological lesions may precede apparent clinical symptoms by a long period of time in the dog. The availability of biomarkers and more refined neuropsychological tests in veterinary clinics may enable the definition of a canine model for mild cognitive impairment in the near future.

We have recently found that the canine is also a suitable AD model from the molecular point of view, because APP and most of the enzymatic machinery for its processing bear extensive homology between dogs and humans (GenBank accessions AY926579 for APP770, AY926580 for APP751, AY926581 for APP714, AY926582 for APP695, AY926587 for ApoE, AY926590 for presenilin-1, AY926591 for presenilin-2, AY926592 for BACE, AY926593 for ADAM-10, AY926594 for ADAM-17 and AY926595 for pleckstrin). In particular, the amino acid sequence of Aβ1-42 is identical in dogs and humans, whereas in rats and mice it differs from the human sequence by three amino acids [26]. Furthermore, the profile of the various shorter Aβ peptides found by mass spectrometry in the canine LCR is almost identical to that found in humans, and both present substantial differences when compared to rats and mice [19]. Thus, the study of both APP and Aβ peptide processing in the dog could help us to understand their biological function and relevance to AD, contributing results more reliably translatable to humans than those obtained using transgenic mice. The dog has also been used in immunotherapeutic assays against fibrillar Aβ, and to confirm observational data in humans that combining an antioxidant-enriched diet and behavioral enrichment (including social, physical, and cognitive components) can lead to substantial improvements in cognition and reduced brain pathology [47, 76].

However, a pitfall for a more extended use of the dog is the dearth of neuroanatomical data regarding the organization, normal aging process, and response to pathological aging of the main neurotransmitter systems implicated in AD. We have begun to study this issue and have made a number of relevant findings. The cytoarchitectural characteristics of the basal forebrain cholinergic neurons in the dog are similar to those of humans, as are several relevant phenotypic characteristics of these neurons, including their expression of NOS, calbindin D-28K, and p75^NTR^ nerve growth factor receptor (Fig. **1D-G**) [77]. As has been found in AD brains, the number of A6-A7 noradrenergic neurons in the dog shows a negative correlation with the extension of cortical Aβ deposits as measured in the gyrus proreus [78].

The investigation of brain aging in the dog also deserves attention from a veterinary point of view. As in the case of human medical technologies, improvements in veterinary medicine and husbandry have contributed to lengthening the lifespan of our domestic animals, with the unwanted side effect of a greater incidence of age-related neurodegenerative diseases [44]. The modern society requires more attention to the welfare of pet animals, increasingly considered as actual “family members”, and there is a growing demand for attention and treatment of these pet dogs’ age related diseases.

## PRIMATES AND DOLPHINS

Primates, like dogs, are among the few animal species demonstrating age-dependent brain pathologies resembling AD [79-82]. An important advantage of several primate models, but not all [80], is that they show AD-like tau-pathology accounting for a more complete disease model. More over, some of these species have relatively short life span as for example the mouse lemur (*Microcebus murinus*) which is considered to be elderly over 5 years of age. Interestingly, among elderly mouse lemurs approximately 20% develop massive brain atrophy, abundant amyloid plaques, cytoskeletal Tau pathology and loss of cholinergic neurons [83]. Furthermore, recently it has been documented the first case of tauopathy with paired helical filaments, ultrastructurally indistinguishable from those seen in Alzheimer's disease, in an aged chimpanzee (Pan troglodytes) [84]. Pathologic forms of tau were histologically identified in neuronal somata, neuropil threads, and plaque-like clusters of neurites throughout the neocortex of this animal. Nevertheless, accumulation of the abnormally phosphorylated tau protein in the brains of animals has been less study than the formation of Aβ deposits. Age-related neurofibrillary changes were described in sheep and goat in the absence of Aβ [85-87] and in wolverine (*Gulo gulo*) [88], polar bear [89] and dog [72] in the presence of Aβ. Thus, non-primate species develop age-related cytoskeletal abnormalities similar to those occurring in humans. However to define an operative model from these species is hampered by which appear to be species specific variations in the process of phosphorylation and cleavage of tau protein. This, together with their phylogenetic proximity to humans, would make primates ideal experimental models (for example, leading studies in immunotherapy are currently being carried out in these species) [90, 91]. However, their use is extremely limited for most laboratories because of high price, restricted availability, maintenance requirements, and ethical considerations.

Other animals that may suffer brain pathology resembling AD are cetaceans. Some cetacean species like dolphins have extended life spans, could be accessible in determined circumstances, and might be useful models to analyze some aspects of the disease. We recently hypothesized that the coastal beaching of these animals could be related to cognitive impairments of the aged individuals (occasionally followed by herds of younger specimens) that might present with Aβ deposits or other AD like neuropathological features. In tissue samples from stranded dolphins, we have found extensive Congo red positive Aβ-immunolabeled deposits throughout the brain, including the cerebellum and the medulla oblongata where such deposits have only been reported in the more severe AD cases^2^. In addition, we have found high amino acidic homology between the APP, BACE, presenilin-1, and presenilin-2 in various dolphin species and their corresponding human counterpart (GeneBank entries: AY926583, AY926588, AY926589, AY926584, AY926586 and AY926585); in particular, the Aβ1-42 peptide in the three dolphin species that have been studied so far (*Grampus griseus, Stenella coeruleoalba *and* Tursiops truncatus*) was 100% identical to the human peptide.

## CONCLUDING REMARKS

There are a variety of species that can serve as experimental models for AD research. In addition to numerous transgenic mouse lines, which have proven to be powerful tools to aid in understanding various aspects of the disease, there are other species that can serve as ‘natural’ wild-type models to shed light on different psychopathological mechanisms or even to try new therapeutic strategies. Among these natural models we emphasize the chick embryo to assay molecules that act on APP metabolism, and the canine model specifically to study the first phase of the disease and to test new therapies. Dogs have a relatively long lifespan (12-20 years depending on the breed), share our environment, and their behavior and clinical state are easier to explore than those of other species. Furthermore, canine APP is identical (98%) to the human counterpart, and dogs are the most accessible among the few animal species demonstrating age-dependent brain pathology and clinical correlates resembling AD.

## Footnotes

1 Domínguez L, Garza V, Lacosta AM, Sorribas V and Sarasa M. Developmental spatiotemporal expression of Alzheimer APP isoforms in the chick embryo. Int J Dev Biol 45(S1): S73-S74 (2001).

2 Sarasa M and Gallego MC. Alzheimer-like neurodegeneration as a probable cause of cetacean stranding. 5th Forum of European Neuroscience. Viena, 8-13th July (2006).
